# Supplementary material for: Defining Populations and Predicting Future Suitable Niche Space in the Geographically Disjunct, Narrowly Endemic Leafy Prairie-Clover (Dalea foliosa; Fabaceae)
Source: Plants (Basel). 2024 Feb 9;13(4):495. doi: 10.3390/plants13040495 (PMC10891826; doi:10.3390/plants13040495)
Supplement: Supplementary file 1 [file plants-13-00495-s001.zip › Morris_et_al_Dalea_PLANTS_Supplemental Figure 1.pdf]

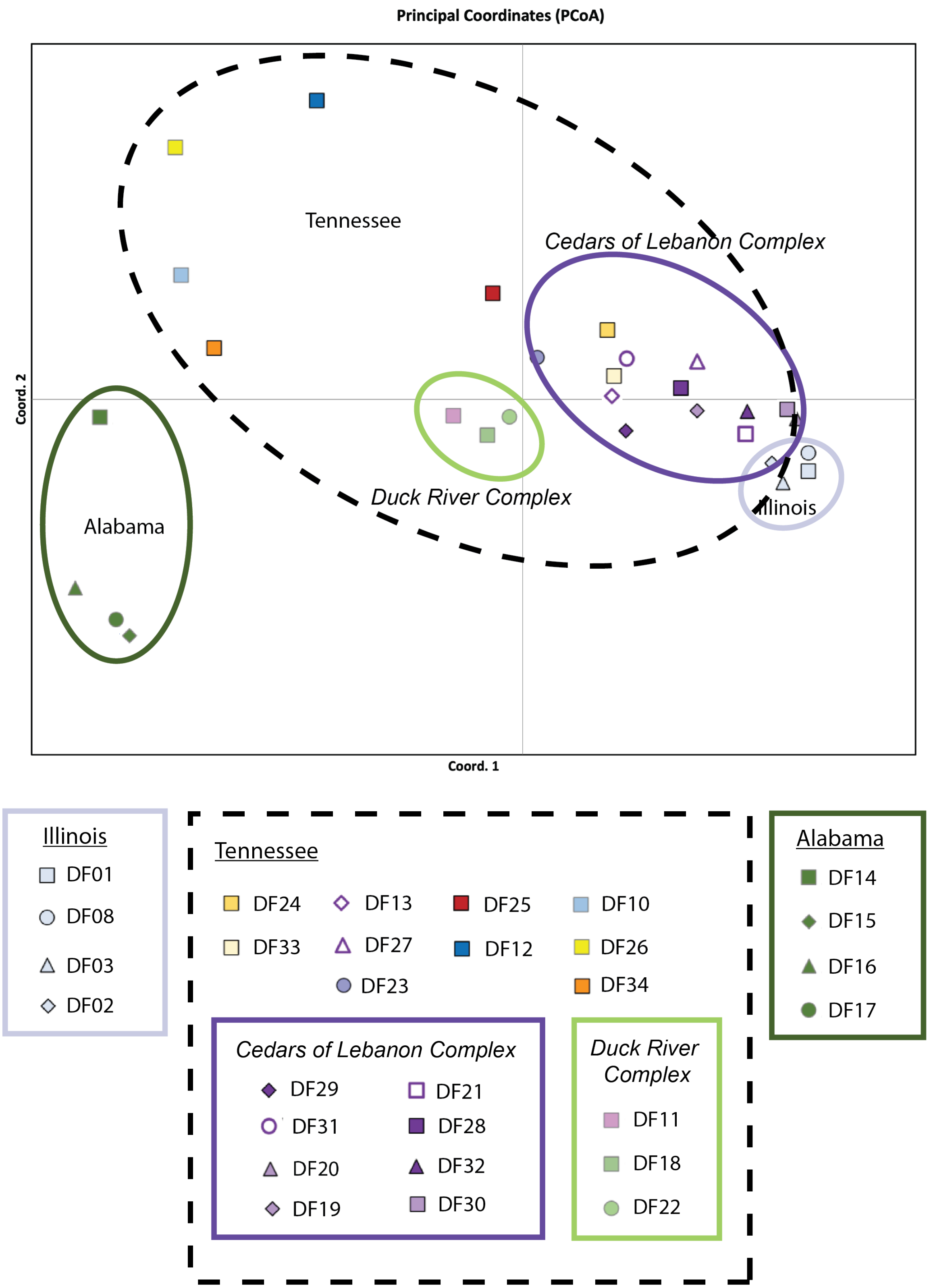

Supplemental Figure S1. Principal Coordinates Analysis (PCoA) of the 29 populations of *Dalea foliosa* sampled across the species range and genotyped for nine nuclear

microsatellite loci. The percentage of variation explained by the first and second axes combined was 50.06%. Color coding is consistent with STRUCTURE clusters presented in Figure 3, with Illinois sites coded in pale blue, Tennessee sites are encircled with a black dotted line and further grouped by management unit (Cedars of Lebanon Complex is encircled in purple, Duck River Complex in light green), and Alabama sites are coded in dark green.
